# Dr. Jenner

**Published:** 1823-03

**Authors:** 


### OBITUARY.

### Dr. Jenner.

We are enabled, through the polite attention of Dr. Baron, of Gloucester, to communicate the following particulars relative to the decease of this distinguished man:

"This most eminent individual was suddenly cut off by an attack of apoplexy, on the morning of Sunday the 21st ultimo, in the 74th year of his age. The night before his seizure, he went to bed cheerful and in apparent health. He rose at his usual hour on Saturday, and came down stairs into his library. Not making his appearance at breakfast, the servant was ordered to go into his room: on doing so, he found his master lying on the floor, the head and upper part of the body being supported on the couch on which he had been sitting. Medical aid was instantly procured, and bleeding and other appropriate remedies were had recourse to without delay. In about four hours thereafter, Dr. Baron, who had been sent for from Gloucester, arrived. Every thing then denoted the rapid approach of the fatal event. The right side was paralysed; the pupil of the eye contracted to a point, and utterly insensible to light; the pulse small, and very irregular; the breathing most distressingly stertorous; and the extremities cold. Efforts were incessantly made to avert the impending blow, but they were quite unavailing, as death took place in about seventeen hours from the first seizure."

Dr. Jenner, in the early part of his career, was destined to the profession of surgery, under the tuition of John Hunter, and afterwards encouraged, by the friendship and patronage of his great preceptor, to practise that branch of the healing art in London. Preferring tranquillity to fame, Jenner declined these flattering prospects, and returned to practise as physician in his native place, Berkeley, little aware how much this determination involved the interests of mankind, or his own individual reputation. He was early devoted to the study of natural history, and wrote an account of the Cuckoo, whose habits were but imperfectly known. This paper is to be found among the Transactions of the Royal Society, of which distinguished body he was soon elected a Fellow. He was the fellow-student, and in after life the friend, of the late Dr. Parry, of Bath, to whom he appears to have communicated some important observations, tending to show the dependence of angina pectoris on organic derangement of the heart. But the greatest of Jenner's discoveries, and the most important pathological fact to be found in the records of medicine, relates to Vaccination. His experiments on the cow-pox were begun in 1797; and, in the following year, with the laudable zeal of a disinterested mind, he communicated to the world a discovery, the concealment of which would have put it in his power to acquire wealth—we may almost say, without bounds. It is not our intention, however, to write a panegyric upon Dr. Jenner, knowing him, as we did, but as a public character. The task of giving his biography, we are happy to find, is in much abler hands: that of handing down his name to posterity, he has left to no one; for, whatever the opinions with regard to the influence of vaccination may be, still, so long as the annals of our art exist, Jenner must hold a conspicuous place among the benefactors of his race.

His remains were deposited in the chancel of the parish church at Berkeley, as privately as possible; but, notwithstanding the wishes of his friends, the concourse who assembled to pay the last tribute of respect to this great philanthropist, was immense. From the general feeling, as well as what passed in the House of Commons on the subject, it is to be presumed that a national monument will be erected to his memory. We observe with pleasure that a meeting was held at Gloucester, on Saturday the 22d ultimo, at which it was resolved to erect a monument in the neighbourhood of that city; the expense of which is to be defrayed by private subscription.

Dr. Baron, of Gloucester, who long enjoyed his confidential friendship, is to become his biographer and the publisher of numerous manuscripts, which are placed in his hands for that purpose. To this work we therefore look forward with confidence, satisfied of the authenticity of the documents, and the talent which will be employed in their arrangement. Meantime we subjoin a reference to some of Dr. Jenner's most important writings:

History of the Cuckoo,—(Philosophical Transactions.)

Inquiry relative to the Origin of the Variola Vaccina.

Observations on the Interference of Herpetic Eruptions with the regular Progress of the Vaccine Vesicle.

Letter to Dr. C. Parry on Artificial Eruptions.

Dr. Jenner likewise published some papers relative to Vaccination in different periodical works. Accounts of these, as they appeared, are to be found in the various Numbers of this Journal, which was established at the time Dr. Jenner's discovery began to attract notice. We refer our readers to volumes i. iii. iv. v. vii. viii. xii. xiii. and xxii.*

The last paper published by Dr. Jenner, and which contains his latest opinions on some points connected with vaccination, is to be found in the Number of this Journal for September 1822.

* Several of the former Numbers of this Journal having been reprinted, any part may now be had by application to the publisher.

